# Clinical significance of non-invasive in vitro drug sensitivity profiling using pancreatic cancer organoids derived from saline flushes collected during routine EUS-FNA

**DOI:** 10.1007/s12094-025-04111-9

**Published:** 2025-11-21

**Authors:** Tomoya Ekawa, Kenji Ikezawa, Yoji Kukita, Makiko Urabe, Yugo Kai, Ryoji Takada, Takashi Akazawa, Yu Mizote, Kumiko Tatsumi, Shigenori Nagata, Hisataka Ogawa, Shinichiro Hasegawa, Hidenori Takahashi, Kazuyoshi Ohkawa, Hideaki Tahara

**Affiliations:** 1 Department of Cancer Drug Discovery and Development, Research Centre, Osaka International Cancer Institute, Osaka, Japan; 2 Department of Hepatobiliary and Pancreatic Oncology, Osaka International Cancer Institute, Osaka, Japan; 3 Laboratory of Genomic Pathology, Research Centre, Osaka International Cancer Institute, Osaka, Japan; 4 Department of Diagnostic Pathology and Cytology, Osaka International Cancer Institute, Osaka, Japan; 5 Nitto Joint Research Department for Nucleic Acid Medicine, Research Centre, Osaka International Cancer Institute, Osaka, Japan; 6 Department of Gastroenterological Surgery, Osaka International Cancer Institute, Osaka, Japan; 7 Centre for Clinical Research, Osaka International Cancer Institute, Osaka, Japan; 8 Department of Cancer Immunotherapy, Research Centre, Osaka International Cancer Institute, Osaka, Japan; 9 Department of Surgery, Graduate School of Medicine, Kyoto University, Kyoto, Japan; 10 Department of Medical Development, Institute for Advancement of Clinical and Translational Science, Kyoto University Hospital, Kyoto, Japan

**Keywords:** Pancreatic cancer, Patient-derived organoids, Drug sensitivity testing, Residual samples from saline flushes, Chemotherapy response prediction

## Abstract

**Purpose:**

To evaluate the in vitro drug sensitivity of patient-derived cancer organoids (PDCOs) established from residual samples from saline flushes (RSSF) collected during routine endoscopic ultrasound-guided fine-needle aspiration (EUS-FNA) in patients with pancreatic cancer.

**Methods:**

Organoids were cultured under mutation-selective conditions. IC_50_ values for 5-fluorouracil (5-FU), SN-38, oxaliplatin, gemcitabine, and paclitaxel were calculated.

**Results:**

PDCOs showed variable responses to the drugs. Exploratory quantitative analysis using ordinal sensitivity scoring and ROC curves (Area under the curve [AUC] up to 0.917) indicated that lower in vitro IC₅₀ scores tended to associate with clinical responders (PR/uPR) compared with non-responders (progressive or stable disease [PD/SD]).

**Conclusions:**

This study demonstrated the feasibility and potential usefulness of in vitro drug sensitivity profiling of pancreatic cancer using RSSF-derived PDCOs. These findings represent the first step toward clinical application, and further validation in larger cohorts is warranted.

**Supplementary Information:**

The online version contains supplementary material available at 10.1007/s12094-025-04111-9.

## Introduction

Pancreatic ductal adenocarcinoma (PDAC) is an aggressive cancer with a 5-year survival rate of less than 10% [1]. Patient-derived cancer organoids (PDCOs) replicate tumor characteristics and can be used to predict therapeutic responses [2]. Previous PDCO studies have primarily used surgical or biopsy specimens to establish drug sensitivity thresholds and correlate them with clinical outcomes. However, comparable analyses using PDCOs derived from residual saline flush samples (RSSF) collected during routine endoscopic ultrasound-guided fine-needle aspiration (EUS-FNA) have not yet been reported. We previously reported a method for generating PDCOs from residual samples from saline flushes during endoscopic ultrasound-guided fine-needle aspiration without additional invasive procedures and achieved an 80% success rate [3]. The present study expands the number of cases and applied genomic and quantitative analyses to explore whether the drug sensitivity profiles of RSSF-derived PDCOs reflected patients’ clinical outcomes. Here, we examined whether the results of in vitro drug sensitivity tests using these PDCOs were associated with the clinical outcomes of patients with or without chemotherapy.

## Methods

### PDCOs and enrolled patients

This study included patients whose PDCOs were successfully established: four from our previous report (3) and three newly enrolled cases; however, one case from the previous cohort was excluded owing to the lack of clinical follow-up data. All PDCOs were established from the RSSF collected before initiating systemic chemotherapy. Informed consent was obtained from all the participants. The clinical histories and treatment timelines of all nine patients, including those excluded from the statistical analysis, are summarized in Supplementary Table S1. This study was approved by the Ethics Committee of Osaka International Cancer Institute (approval no. OICI 18118).

### *Establishment and *in vivo* transplantation of PDCOs*

PDCOs were established from RSSF collected during routine EUS-FNA and cultured in Matrigel using mutation-selective media for KRAS, SMAD4, or TP53, as previously described [3]. The descriptor following each patient ID in Fig. 2 (e.g., “P1–KRAS”) denotes the driver mutation used for the mutation-selective medium when establishing the PDCO. Seven PDCOs were successfully generated from nine patients (77.8%), while two were discarded because of bacterial contamination after the initial organoid formation. P2-derived PDCOs were transplanted subcutaneously into NOD.Cg-*Prkdc*^*scid*^/J (NOD/SCID) mice (Jackson Laboratory) and the tumors were excised, dissociated, and re-cultured to assess the stability of the mutation profiles.

### Gene mutation analysis of PDCOs

Genomic DNA was extracted using the QIAamp DNA Mini Kit (Qiagen, Hilden, Germany) following the manufacturer’s protocol. Mutational analysis was performed using TruSight Oncology 500 (TSO500, Illumina), which detects single-nucleotide variants (SNVs), indels, and structural variants across 523 cancer-related genes [4].

### In vitro drug sensitivity tests using PDCOs

The organoids were dissociated into single cells and seeded at 1000 cells/well in 96-well plates. After 3 days, 5-fluorouracil (5-FU), SN-38, oxaliplatin, gemcitabine, and paclitaxel were added at the indicated doses and incubated for 72 h [5]. Cell viability was measured using the RealTime-Glo™ MT Cell Viability Assay (Promega, Madison, USA). The IC_50_ values were calculated using GraphPad Prism version 8.4.3. Drug sensitivity was interpreted by comparing individual IC_50_ values with the median IC_50_ of the entire cohort.

### Statistical analysis

Of the seven patients from whom PDCOs were successfully established (ten lines in total), clinical follow-up was available for six (Tables 1, 2). For the quantitative correlation analysis, patients P2 (postoperative adjuvant chemotherapy) and P5 (second-line TS1 regimen; recurrent disease) were excluded, leaving five patients for analysis (Table S1).

**Table 1:**
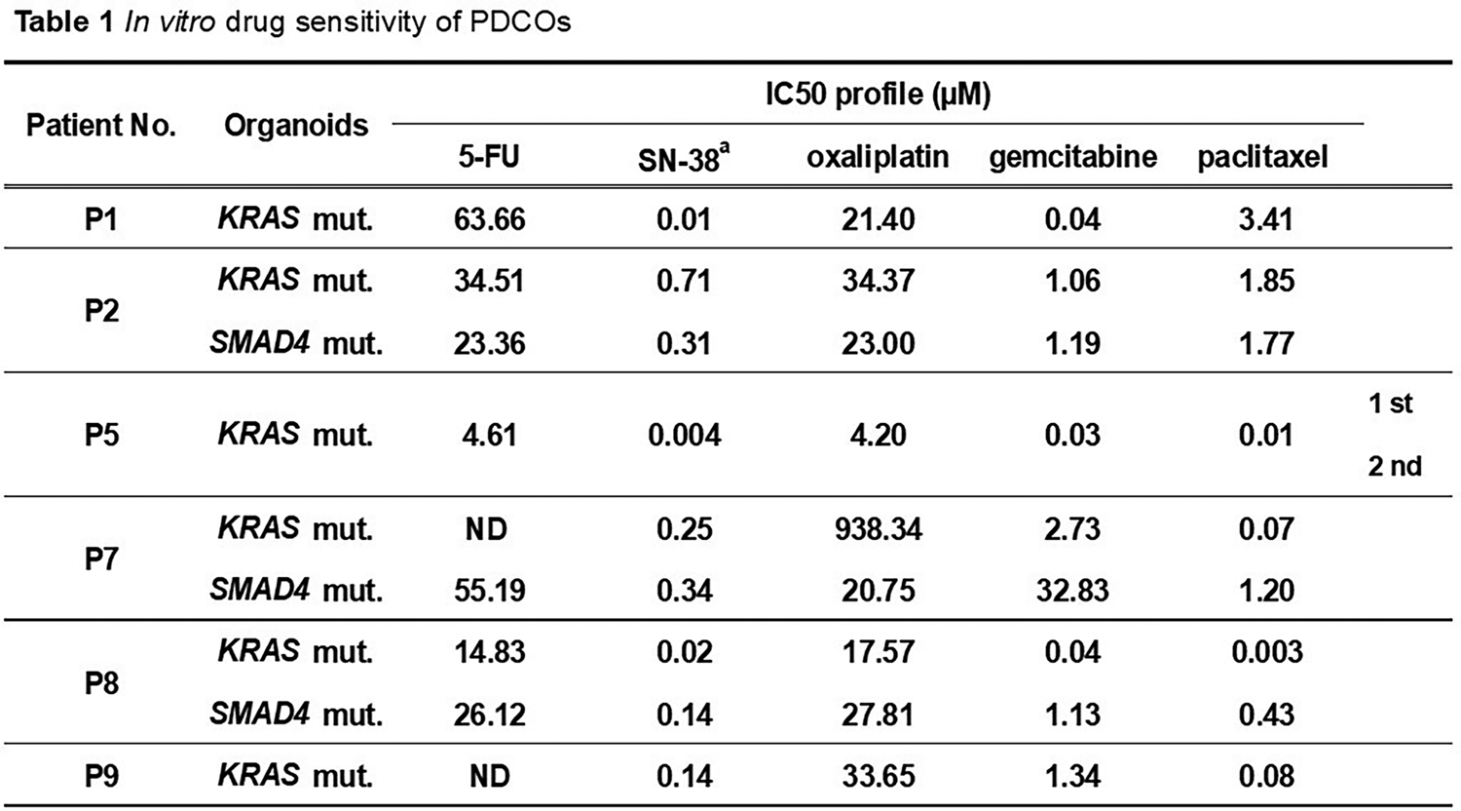
In vitro drug sensitivity of PDCOs

^a^SN-38: Active metabolite of irinotecan

**Table 2:**
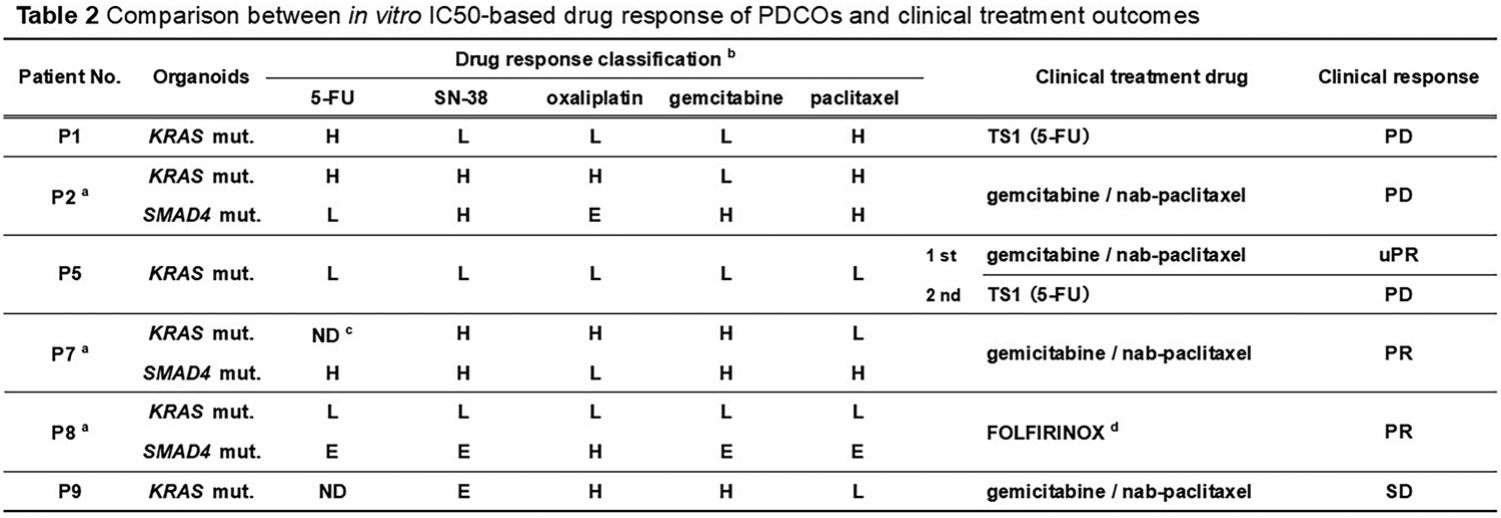
Comparison between in vitro IC_50_-based drug response of PDCOs and clinical treatment outcomes

^a^Two PDCO lines were established from patients P2, P7, and P8

^b^Drug sensitivity: Based on IC_50_ relative to the median IC_50_ of the nine PDCO lines tested. H = high IC_50_ (low sensitivity), L = low IC_50_ (high sensitivity), E = IC_50_ near the median (intermediate sensitivity)

^c^ND: Not detectable

^d^FOLFIRINOX: Combination of 5-FU, leucovorin, irinotecan (SN-38), and oxaliplatin

Based on Table 2, the drugs administered to each patient were identified, and the organoid-level IC_50_ values were converted into ordinal sensitivity scores (high = 2, equivalent = 1, low = 0; Table S1). For each patient, scores were integrated under three assumptions (Min, Mean, and Max) to represent the most sensitive, average, and most resistant clones, respectively. Associations between integrated scores and clinical responses (PR/uPR = 1, progressive or stable disease [PD/SD] = 0) were evaluated using the Mann–Whitney *U* test and receiver operating characteristics/area under the curve (ROC/AUC) analysis. A one-sided alternative was pre-specified a priori based on the hypothesis that responders would show lower ordinal scores than non-responders. As the sample size was very small and model instability (e.g., quasi-complete separation) was a concern, logistic regression was not interpreted. The statistical results are summarized in Supplementary Table S2.

### Clinical assessment of patients with PDAC

Tumor responses were evaluated based on radiographic tumor size using RECIST v1.1 criteria.

## Results

**Fig. 1:**
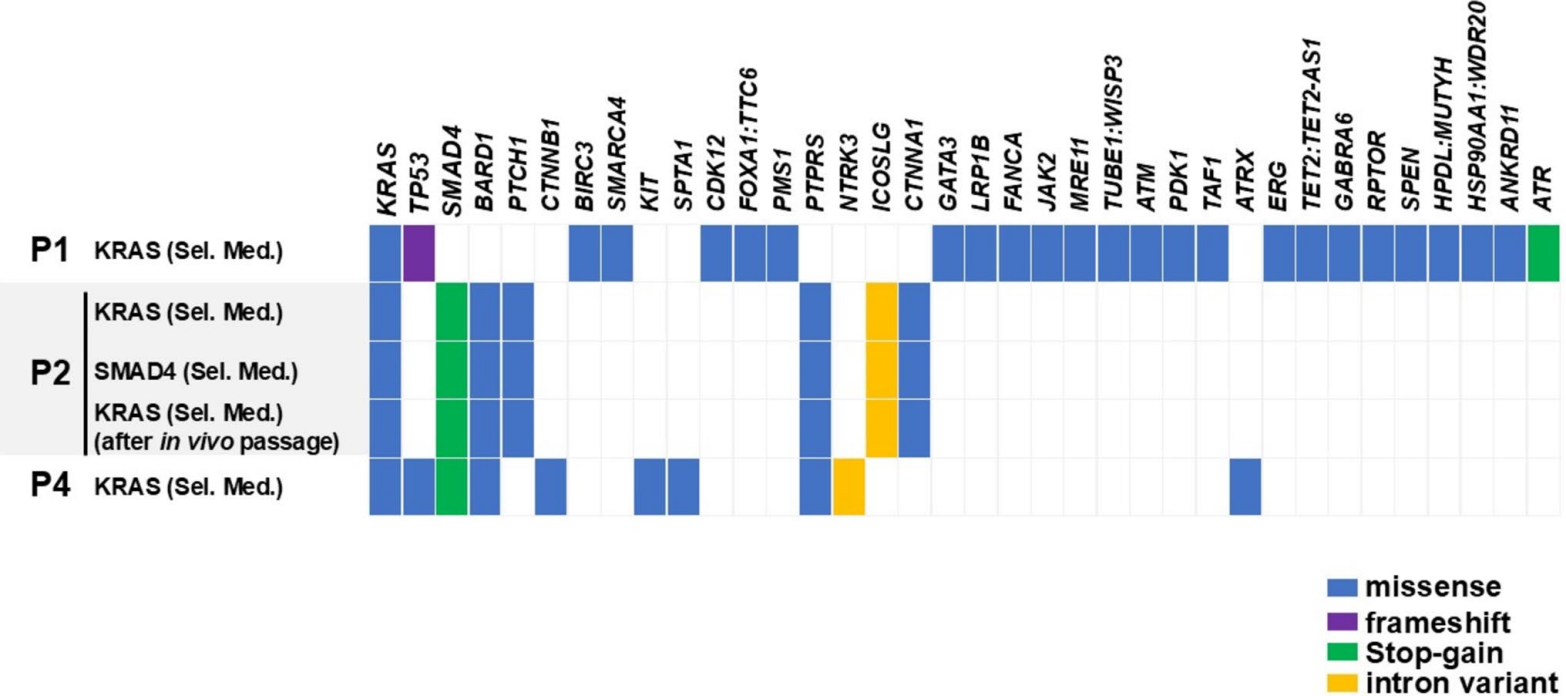
Genetic mutation profiles of PDCOs analyzed using TSO500. Somatic mutations in the PDCOs from P1, P2, and P4 were identified using the TruSight Oncology 500 (TSO500) panel. The mutation types included missense, frameshift, stop-gain (nonsense), and intron variants. Labels, such as “KRAS (Sel. Med.)” and “SMAD4 (Sel. Med.)”, show the culture conditions under which each PDCO was derived “Sel. Med.” Refers to a selective medium designed to promote the growth of tumor cells harboring specific mutations (for example, in KRAS or SMAD4)

The PDCOs analyzed were maintained in mutation-selective medium for ≥ 5 passages (median 44–112 days, corresponding to the time required for five passages among ten PDCO lines established from seven patients), during which no motile or macrophage-like cells were observed during routine microscopic inspection, supporting that the cultures consisted predominantly of epithelial tumor cells. Using the TSO500 panel, the presence of canonical PDAC driver mutations (*KRAS*, *TP53*, *SMAD4*) was confirmed in the examined PDCOs. These findings are consistent with previous reports that pancreatic cancer organoids preserve the genetic and phenotypic features of their original tumors [6, 7]. Because healthy tissues or unrelated cell line controls were not feasible with RSSF, drug sensitivity was interpreted relative to cohort medians and restricted to drugs actually administered clinically. Analysis using the TSO500 panel revealed that the PDCOs from P1, P2, and P4 harbored KRAS missense mutations (G12C or G12D). P2 also carried a SMAD4 nonsense mutation (C115X). For P2, the PDCOs derived from both KRAS and SMAD4 mutation-selective media exhibited the same mutation profile, suggesting that a genetically identical tumor cell population harboring both mutations was enriched under each condition. Although TP53 mutations were detected in PDCOs from P1 (frameshift) and P4 (missense) using TSO500, these organoids did not grow in TP53 mutation-selective medium, possibly because of the low abundance of TP53-mutant cells or a lack of selective growth advantage. Similarly, although a SMAD4 nonsense mutation was identified in PDCO from P4, it failed to expand in the SMAD4 mutation-selective medium, potentially owing to insufficient representation of SMAD4-mutant cells. Comprehensive genomic profiling using the TruSight Oncology 500 panel (523 genes) was performed on representative PDCOs, and all detected variants are presented in Fig. 1. For clarity, genes without any detected alterations are not listed.

In addition to *KRAS* and *SMAD4*, multiple genetic alterations were identified across the panel, including variants of genes, such as *BARD1*, *PTCH1*, *CTNNB1*, *BIRC3*, *SMARCA4*, *KIT*, and *CDK12*. These variants included missense mutations, frameshifts, and splice-site alterations (Fig. 1). Because the mutation profile of in vivo passaged organoids (P2) remained consistent with that of the original in vitro PDCOs, the PDCOs were genetically stable, even after in vivo proliferation.

**Fig. 2:**
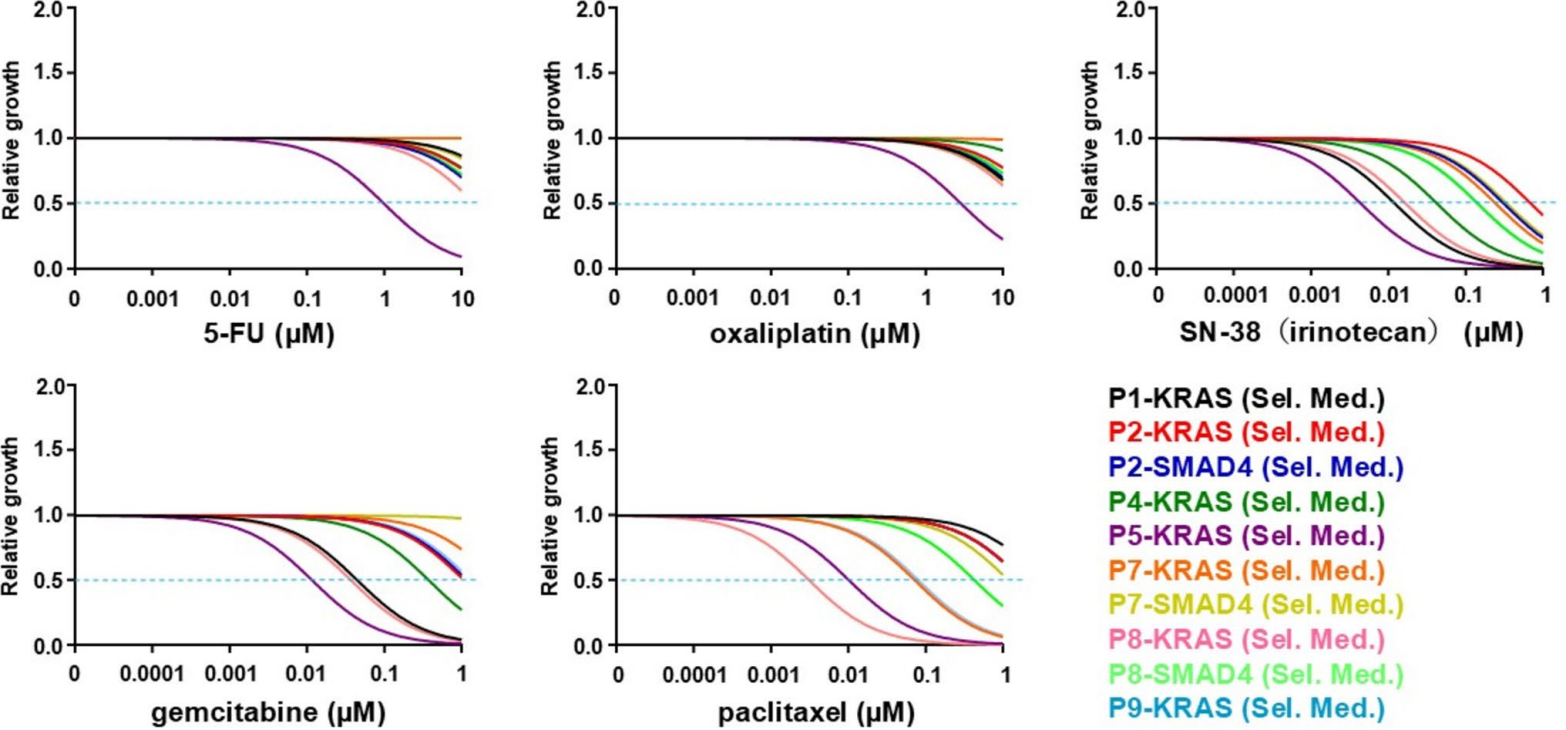
Dose–response curves of PDCOs established from RSSF in response to five chemotherapeutic agents. Cell viability was normalized to that of untreated controls. Each curve represents the organoid line grown under mutation-selective conditions. The legend lists patient IDs; the genetic label next to each ID (e.g., “P1–KRAS”; “Sel. Med.”) indicates the driver mutation used for the mutation-selective medium when establishing the PDCOs

The in vitro drug sensitivity profiling of ten PDCOs established from seven patients, based on cell viability, with IC50 as the primary readout [6], while acknowledging that viability-based organoid pharmacotyping frameworks are widely used [12, 13], revealed considerable inter-patient and inter-drug variability (Fig. 2; Table 1). Figure 2 shows the dose–response curves of all ten PDCO lines derived from seven patients (P1–P9), illustrating the range of drug sensitivities observed. The IC_50_ values for each of the five standard chemotherapeutic agents varied across the PDCOs. This may reflect patient-specific drug responses. For example, the PDCO from P1 showed a markedly high IC_50_ value of 63.66 µM, suggesting low sensitivity to 5-FU, whereas the PDCOs from P5 exhibited uniformly low IC_50_ values across all tested agents, consistently suggesting high sensitivity to them.

Using Table 2 as a reference for clinically administered regimens, the organoid IC_50_ values were converted into ordinal sensitivity scores (high = 2, equivalent = 1, low = 0; Table S1) and aggregated into patient-level minimum, mean, and maximum scores. Patients in P2 and the P5 second-line therapy groups were excluded from the analysis (Table S1). Exploratory ROC/AUC analyses yielded an AUC of 0.833–0.917, which was the highest for the Min score, indicating lower IC_50_-derived ordinal scores for PR/uPR than for PD/SD (Table S2). PDCOs from patients who were evaluated as having a partial response (PR) (P5 1 st, P7, and P8) or stable disease (SD) (P9) tended to have lower IC_50_ values for the agents used in the treatment. In contrast, patients with progressive disease (PD) (P1 and P2) typically had PDCOs with higher IC_50_ values. These results suggest that the PDCO-based assay may have potential as a predictive tool for the chemotherapy responsiveness of tumors in patients.

## Discussion

PDCOs were established from RSSF using mutation-selective culture media, which enabled the selective growth of cancer cells while minimizing contamination by normal epithelial precursors [6]. They retained key driver mutations, such as *KRAS* and *SMAD4*, and additional mutations were identified in *PTCH1*, *BARD1*, *CTNNB1*, *SMARCA4*, and *CDK12* (Fig. 1). Furthermore, identical mutations were detected in the P2-derived PDCOs after in vivo passaging [7]. These results demonstrate that PDCOs retain canonical driver mutations and a broader spectrum of genomic alterations, suggesting that they can be used as samples for comprehensive molecular profiling.

Notably, although tumor tissue-based gene panel testing is increasingly performed in clinical practice, its success rate is suboptimal (for example, 57% for EUS-FNA samples in Japan) [8]. In addition, there are concerns regarding the risk of needle tract seeding due to EUS-FNA; therefore, minimizing the number of punctures is desirable [9]. Our data suggest that RSSF-derived PDCOs, which do not require additional invasive sampling, may serve as an alternative source for genomic profiling, particularly in cases with limited tumor tissue.

PDCOs also demonstrated patient-specific responses to chemotherapeutic agents (Fig. 2, Table 1), consistent with prior findings [10, 11]. Because absolute cutoffs for sensitivity could not be determined owing to the small sample size in our study, we classified sensitivity based on whether the IC_50_ values were above or below the cohort median. Table 2 shows the relationship between in vitro drug sensitivity and clinical responses. Although the study was underpowered to detect statistical significance, exploratory analyses using ordinal scoring and ROC curves (AUC = 0.833–0.917; highest for the Min score) suggest that lower in vitro IC₅₀-derived scores are associated with clinical partial response (PR/uPR) rather than PD/SD. These results support the clinical relevance of organoid-based readouts and motivate prospective validation in larger cohorts. Supplementary Table S3 summarizes the mean IC_50_ values (± SD), coefficients of variation, and ranges for all five drugs tested across the PDCO panel. Compared with previously reported IC_50_ ranges in established pancreatic cancer cell lines (e.g., gemcitabine 0.3–58 µM; 5-FU 18–96 µM [14, 15]), some of the values were higher or lower, which may reflect inter-patient biological diversity of patient-derived PDCOs. This indicates that the PDCOs from patients with partial responses (P5, P7, and P8) exhibited higher sensitivities to the administered drugs, whereas those from patients with disease progression (P1 and P2) showed lower sensitivities.

This study followed our previous study [3] and defined PDCO establishment as achieving ≥ 5 passages (44–112 days across 10 lines from seven patients) to ensure stability. Nevertheless, visible colonies formed within 7–14 days, and drug testing was initiated after 2–3 passages (approximately 3–5 weeks). This suggests that PDCO-based assays may provide timely information for clinical decision making in selected cases although further validation is required before routine implementation. However, evolutionary changes after prior therapy, including drug-induced clonal selection, may attenuate the predictive value of pre-treatment PDCOs and should be considered in their interpretation. As this study was designed to demonstrate a non-invasive IC_50_-based assay, systematic expression assays of drug resistance genes, such as *ABCB1/MDR1* and *ABCG2,* were not performed. Importantly, PDCOs were generated from RSSF obtained during routine EUS-FNA without requiring additional invasive procedures, which might be disadvantageous for patients. This approach preserves tumor-specific mutations, allows in vitro drug screening, and reflects clinical outcomes in multiple cases. Although further validation in larger cohorts is needed, our results highlight the feasibility and clinical potential of this strategy as a platform for personalized therapy of pancreatic cancer.

## Conclusion

Our results demonstrate the feasibility of using PDCOs derived from RSSF for in vitro drug sensitivity profiling of pancreatic cancer. This approach may be a powerful tool for the development of better precision medicine. However, further validation in a larger cohort is required.

## Supplementary Information

Below is the link to the electronic supplementary material.

Supplementary file1 (JPG 105 KB)

Supplementary file2 (JPG 108 KB)

Supplementary file3 (JPG 89 KB)

## Data Availability

The datasets generated and/or analyzed during the current study are not publicly available because of privacy or ethical restrictions, but are available from the corresponding author upon reasonable request.
