# Supplementary figures and images for: Clinical significance of non-invasive in vitro drug sensitivity profiling using pancreatic cancer organoids derived from saline flushes collected during routine EUS-FNA
Source: Clin Transl Oncol. 2025 Nov 21;28(5):1940–6. doi: 10.1007/s12094-025-04111-9 (PMC13099696; doi:10.1007/s12094-025-04111-9)

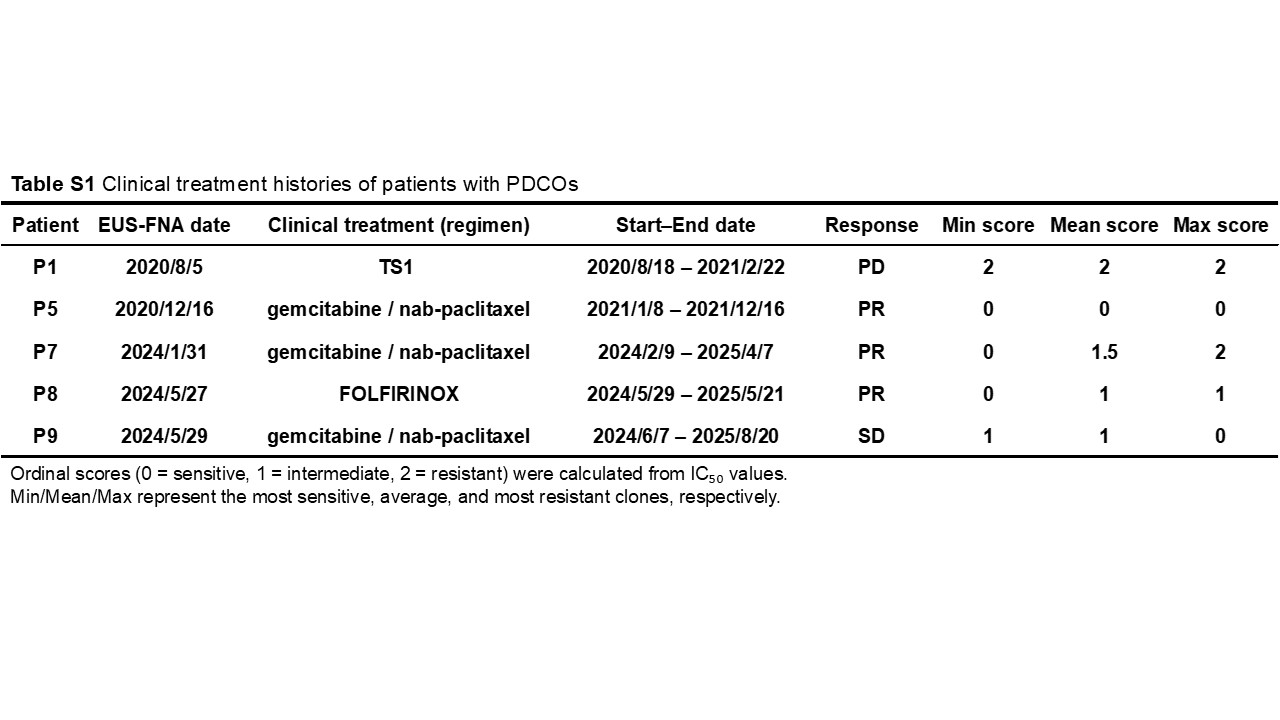

Supplement: Supplementary file 1 — Supplementary file1 (JPG 105 KB) [file 12094_2025_4111_MOESM1_ESM.jpg]

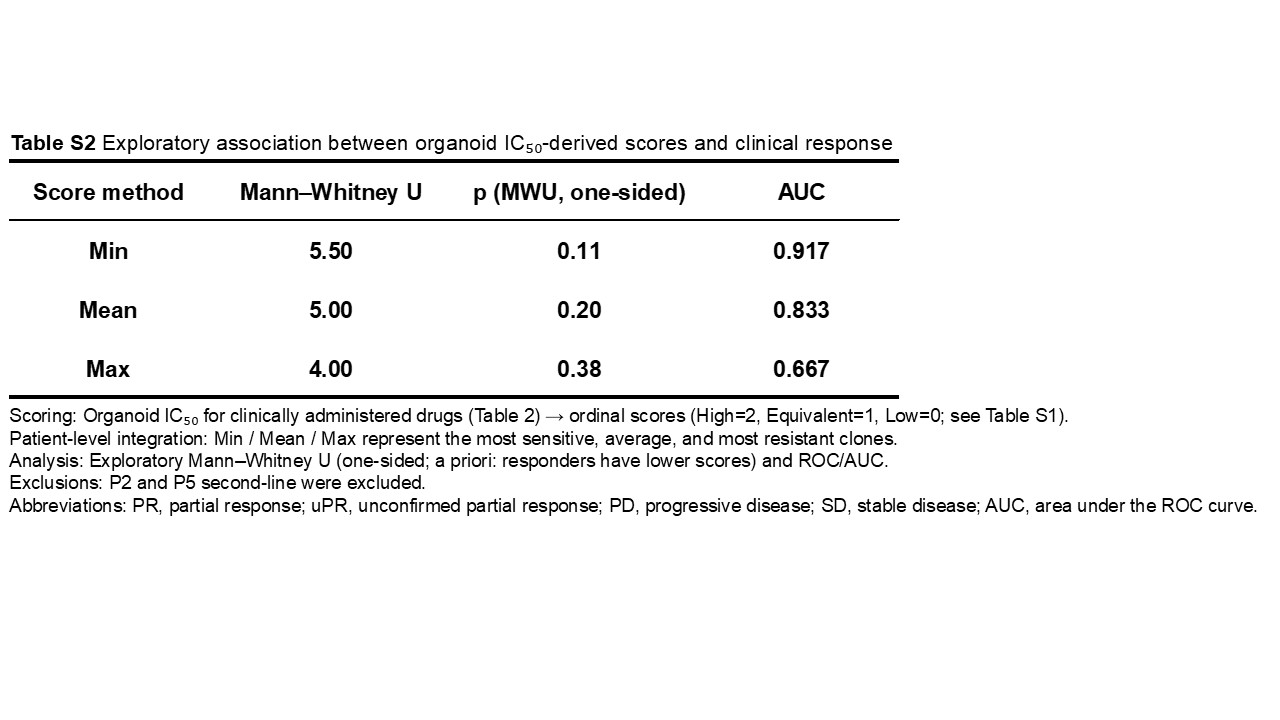

Supplement: Supplementary file 2 — Supplementary file2 (JPG 108 KB) [file 12094_2025_4111_MOESM2_ESM.jpg]

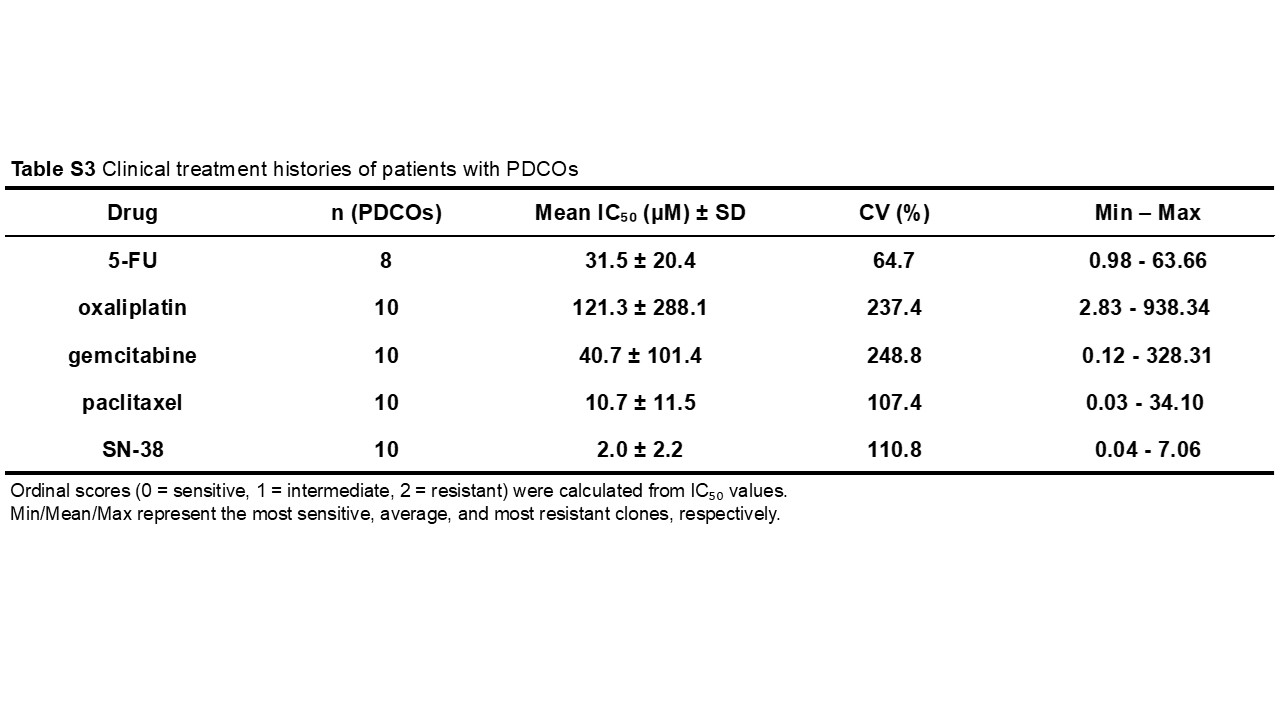

Supplement: Supplementary file 3 — Supplementary file3 (JPG 89 KB) [file 12094_2025_4111_MOESM3_ESM.jpg]
